# Financial risk protection and Nigeria’s journey towards universal health coverage

**DOI:** 10.1038/s44401-025-00021-8

**Published:** 2025-05-21

**Authors:** Nkaiso Lawrence Essien

**Affiliations:** Department of Pharmacy, The Good Shepherd Specialist Hospital, Enugu, Nigeria

**Keywords:** Health care economics, Health policy

## Abstract

Financial risk protection is essential to the attainment of universal health coverage in Nigeria. Using narrative synthesis, this paper shows how ineffective implementation of health financing mechanisms birth unfavourable financial risk outcomes. It is imperative that focus shifts from pooled payment systems to tax-based funding for health in Nigeria’s informal sector driven economy. Meanwhile, political will must be mobilized to optimize the social health insurance program’s protective effects from financial risk.

## Introduction

Over the last two decades, the intensity of public health interventions aimed at achieving universal health coverage (UHC) in Nigeria has increased as occasioned by the revamping of the primary healthcare service, restructuring of the social health insurance program and introduction of the National health workforce registry (NHWR)^1–3^. According to the World health Organization (WHO), UHC means that every healthcare user has access to all the quality services they need when they need them without incurring financial hardship^4^. This requires the collaboration of multisectoral stakeholders within and outside the country in line with the sustainable development goals of the United Nations. Currently, the UHC service coverage index for Nigeria is 38, showing a trend reversal of consistent improvement over the last 20 years while being significantly lower than the continental and global averages^5^. Although there was a global decrease in service coverage between 2019 and 2021, the decline was steeper on the African continent and in Nigeria, highlighting a major disparity in the burden of the COVID-19 pandemic^6–8^. Further review of the UHC situation shows stagnation in financial risk protection despite implementation of relevant policies and interventions. Financial risk protection (FRP) is the goal of UHC which ensures that direct payments for healthcare do not cause financial hardship or affect living standards^9^. This neglect has significantly truncated her advancement towards UHC.

Given that UHC is a multifactorial concept comprising population coverage, service coverage, and financial risk protection, there are numerous scholarly debates on which tools are efficient in evaluating the overall service index^10,11^. However, in the WHO global health observatory there are indicators of UHC service coverage index, which are subdivided into health service coverage (measured under 4 components) and financial hardship caused by healthcare payments^6^. These indicators show underperformance in both categories which echoes the broad-spectrum inefficiency of the national health system underscored by several political and socioeconomic factors^12^. However, it has been posited that for UHC to be achieved, there is a need for separate evaluations of the various indicators and dimensions. Ataguba and Ingabire^13^ argued that in the assessment of UHC, population coverage and financial risk protection are often downplayed, with more focus given to service coverage. This notion is supported by other scholars who cite limited evidence on key indicators^14^. Beyond measuring mechanisms, there is still a debate on the conceptualization of financial risk protection and the design of effective interventions^15^. These factors all contribute to the futility of attaining UHC in Nigeria.

To this end, this article was aimed at broaching the subject of FRP in the Nigerian context. It analyzed the progress in the FRP discourse on the basis of scholarly contributions to the available literature. It also reviews relevant policies and strategies implemented and evaluates their strengths and limitations. This enabled the identification of reasons for suboptimal outcomes regarding UHC and suggestions for improvement.

## UHC And FRP In Nigeria – Small Beginnings

Peter Sands^16^ noted Nigeria’s vast population, wide burden of disease (communicable and non-communicable) and poverty as the necessitating factors for the development of interventions aimed at improving UHC outcomes in the nation. The concept of UHC originated from the 1948 WHO constitution, which confers the status of human right to “healthcare” with the goal of making it attainable to all^17^. Initially, UHC focused on the provision of primary healthcare services, especially within vulnerable healthcare systems such as Nigeria. The WHO also mandated that all member states follow this roadmap, as enshrined in the 1979 Alma-Ata declaration and later the Ottawa Charter for health Promotion^18^. Taking baby steps, Nigeria adopted the National Basic Health Sciences Scheme in 1970. Although largely ineffective due to poor financing, the program saw a sharp upturn in fortunes under the auspices of Prof. Olikoye Ransome-Kuti who scaled Primary Healthcare Center (PHC) coverage to all local governments in Nigeria^19^. This effort focused on preventive health, reproductive/maternal and child health and immunization. Measurable successes were achieved with immunization coverage increasing to 80%. However, progress was truncated by the transition to a military regime in the 1980s which effectively stripped powers from local governments who had been granted governance over the program^20^. Following Nigeria’s return to democratic governance in 1999, the newly appointed minister of health, Eyitayo Lambo on his agenda prioritized, among others, a revamp of the primary healthcare structure and improvement in financing^21^. This is on the grounds that proper health financing is a predictor of affordability and uptake of the available healthcare services. Following this recognition, in 2005 the first National Health policy (NHP) since 1988 was developed to guide further efforts towards the attainment of UHC^22^. This policy sought to expand pooled financing options to include the private sector and the engagement of communities in prepayment efforts. Most notably, they sought to increase government spending on healthcare to be on par with international standards. Under the structural adjustment program of the military regime, government spending on healthcare, which was already under 10%, was further reduced^23^. Additionally, most health institutions were privatized and private payments became the norm, taking the nation further away from its UHC goals. At the time of its development, government spending on healthcare as a percentage of total expenditure was between 4% and 6%^24^.

**Fig. 1:**
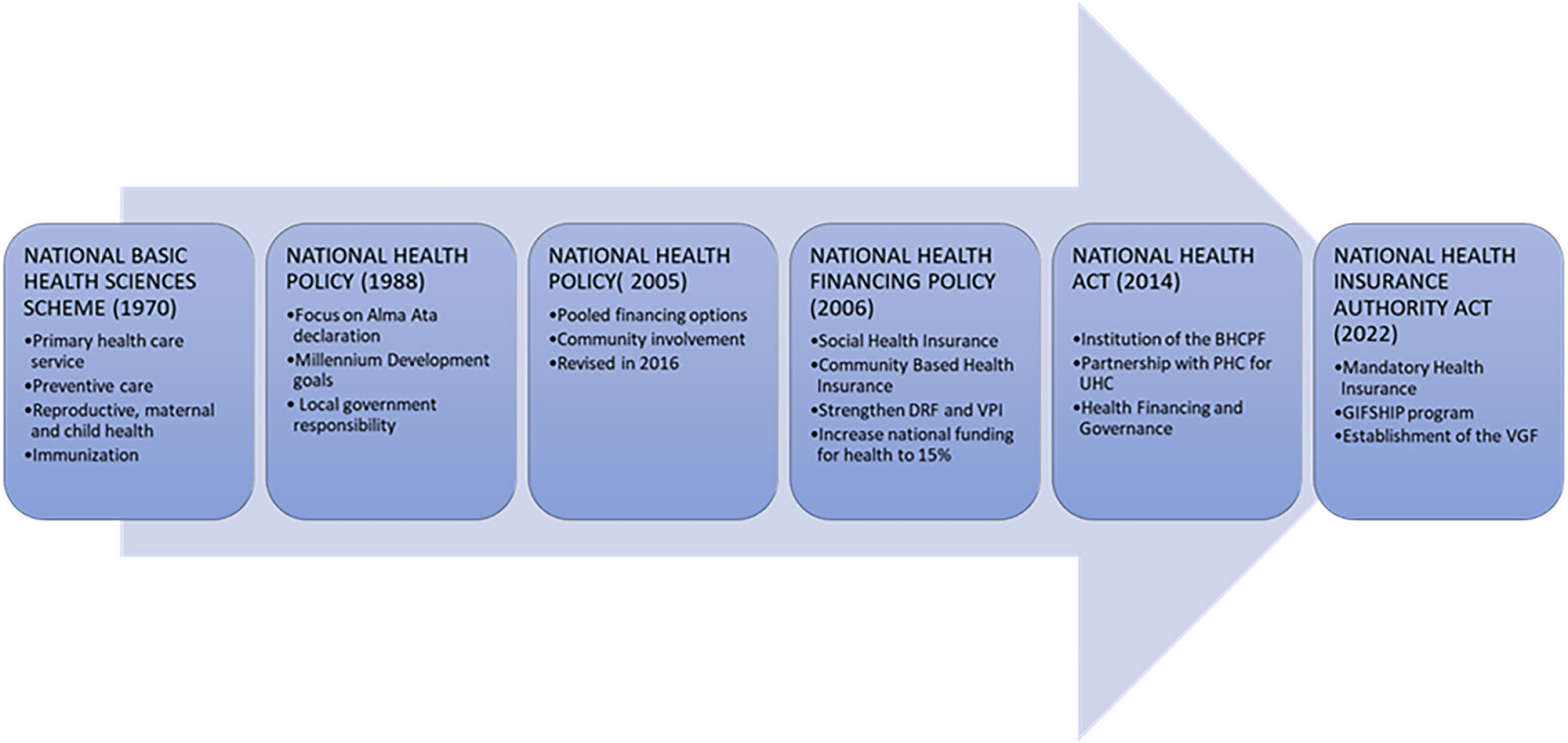
Chronology of relevant health financing policies in Nigeria. The section “UHC AND FRP IN NIGERIA – SMALL BEGINNINGS” presents the relevant policies influencing health financing in Nigeria between 1970 and 2022 in a chronological order. This figure summarizes the contents of that section into an image for better understanding. Each blue curved rectangle carries a specific policy, with the main features of that policy being highlighted in bullet points. The blue arrow in the background shows the direction of progression.

Following the NHP, the more targeted National Health financing policy (NHFP) was developed in 2006, which focused on achieving universal health coverage with financing risk protection at its core^25^. Its main goals were to see that funds are made available and that these funds are efficiently allocated and made available for mandated purposes. Under this policy, Community-Based Health insurance (CBHI) and Social Health Insurance (SHI) schemes were established, which sought to increase coverage to the 70% of citizens outside the public service system uz^26^. It also mandated that governments at all tiers increase funding for health above 15% of the total budget^27^. In addition, it established support for existing health financing programs such as drug revolving funds, voluntary private insurance and state-owned insurance schemes. With these two policies in place, Nigeria was ultimately on the path towards achieving universal health coverage. Figure 1 presents these policies in chronological order. Subsequently, the Nigerian government would revise the NHP in 2016 to accommodate evolving realities in the health landscape like pandemics, insurgency and climate change.

In 2014, the National health act was developed to majorly addresses shortfalls in health financing and governance^28^. The most important provision of this act was the establishment of the Basic health Care provision fund (BHCPF). This meant the allocation of 1% of the national consolidated revenue along with contributions from international and domestic partners for the delivery of the basic minimum package of healthcare services and emergency medical treatment^29^. This fund supports vulnerable groups, covers essential medicines and vaccines and enhances access to these services for providing transportation for primary healthcare needs. To achieve this, 45% of the BHCPF was disbursed to the national primary health care development agency (NPHCDA) to strengthen PHC services^29^.

PHC is integral to the success of UHC efforts as it enables wider coverage (including rural communities) and eliminates socioeconomic inequities. Specifically, PHC improves FRP by lowering health costs and reducing length of admission for the patients, and through its integration with public health, reduces overall health expenditure, thereby reserving funds for injection into the pooled payments system^30,31^. The National Health Insurance Scheme (NHIS) would also leverage this for the expansion of service delivery and coverage through the SHI schemes^32^. Public PHC facilities were co-opted as main service points coverage of the 9565 wards in all 774 local government areas. This eliminated barriers to access like distance and transportation costs and language barriers^33^. Other financial risk benefits of this move include the cost containment as the BHCPF allocations ensured that people in underserved areas and lowest socioeconomic quintiles can assess healthcare without having to pay at point of service^29^. However, dissatisfaction with the NHIS by service users, inconsistent remuneration to healthcare providers and other challenges would necessitate the development of the National Health Insurance Authority (NHIA) act in 2022^34^.

## The FRP Discourse – Current Realities

Despite the lofty ambitions of the NHP, the NHFP and the ensuing interventions, Nigeria remains far from attaining defined targets. Uzochukwu and colleagues^26^ extensively discussed unmet needs with health financing. They showed an enormous gap in financing, with the federal government going back on its initial 15% pledge to a meager 6% allocation for health expenditure^27^. The most recent data from the WHO indicate that domestic government healthcare expenditure was at 4.1%^24^. This exacerbates the dependence on out-of-pocket payments (OOP) for health needs, thereby exposing users to financial hardship^35^. Although FRP is often measured via two indicators, catastrophic health expenditure and impoverishing health expenditure, Rahman and others^36^ revealed additional indicators (adoption of strategies to cope with healthcare and forgone healthcare), which provide a robust view of the burden of financial risk borne.

## Catastrophic Health Expenditure (CHE) And Impoverishing Health Expenditure (IHE)

**Fig. 2:**
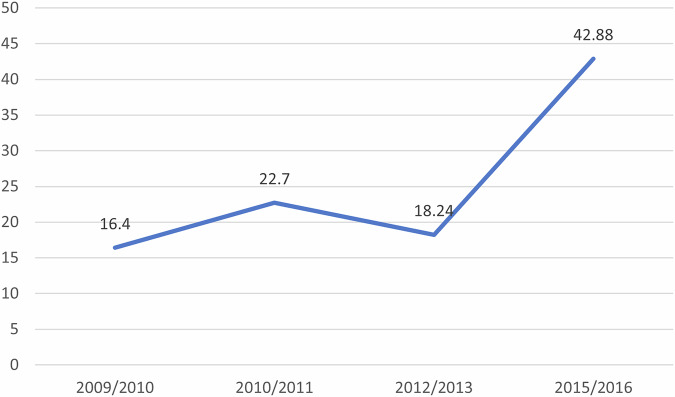
Incidence of CHE in Nigeria at 10% of total household expenditure. The figure shows the trend in national incidence rates for CHE in Nigeria between 2010 to 2016. In this chart, CHE is measured at 10% of the total household expenditure. Data for the chart was obtained from studies that utilized secondary data from the Harmonized Nigeria living standard survey in 2009/2010 and the Nigeria general household surveys in 2010/2011, 2012/2013 and 2015/2016 respectively^14,38^. It shows an increasing trend pattern after a short decline between 2011 and 2013. The values highlighted on the blue line show the incidence of CHE in each corresponding year.

According to the World Bank 2020, 3.5% of Nigerians were impoverished due to OOP. IHE is also a determinant factor in the multidimensional poverty of the nation^37^. In 2019, incidence of CHE was 56.33% at 10% of total household (non-food) expenditure (THE) and 27.02% at 25% of THE^38^. With the worsening of economic realities in the region, there has been an upwards trend in the share of households facing CHE as shown in Fig. 2^39^.

Ataguba argues that lack of consensus on what constitutes catastrophic expenditure among scholars invariably affects the development of adequate policies that can address the inequitable variance of the impact of OOP on families and individuals in various socioeconomic quintiles^40^. This affects the comparison of various reports based on the threshold of measurement. Other studies argue against the use of non-disease or nonservice-specific thresholds to access financial risk protection as some families in the lower quintile may face financial risk at the generalized 40% or 10% thresholds^41,42^.

This high incidence of CHE holds true for non-communicable diseases (NCDs), as researchers report that 30% of households with NCD incurred CHE at the 40% capacity-to-pay (CTP) threshold. A further 20% were pushed below the poverty line by these healthcare costs with 10% being on the verge of impoverishment^43,44^. However, they reported a higher level of CHE among patients who used private healthcare facilities. Nonetheless, this variance was not sufficient to drive impoverishment. For cancer and sickle cell disease, CHE levels are much higher than the general averages due to the higher cost of drugs and frequency of hospitalization, which inflates both direct and indirect costs ^45,46^.

Using Enugu as a focal point, Okafor et al.^47^ reported that the incidence of CHE was 36% among patients with Diabetes Mellitus, 31.1% for hypertensive patients and 31.1% for those who were living with comorbidities at a threshold of 10% of income. In follow-up surveys, the incidence of CHE among diabetes patients increased, but that of hypertension patients reduced. Another study on the Uyo population revealed similarities in the average monthly cost for the management of hypertension ($20.43 versus $27.9)^48^ However, their study differentiated costs incurred due to hypertension alone from other costs associated with other comorbidities. They also explored various coping mechanisms which included received external financial support for treatment or transportation or paying out of pocket for either only transportation or treatment as supported by other studies^47,49^.

There is a need for a revalidation of some of these numbers as the exit of multinational pharmaceutical companies like GSK from the Nigerian health space has led to a spike in the cost of medicines and other medical supplies^50^. This is due to the reduced competition for other manufacturers, supply chain disruptions that lead to scarcity of these products^51^. Considering that medication costs constitute a significant proportion of health costs, it is expected that CHE and IHE levels will be further increased.

Conversely, CHE rates are very low for communicable diseases such as measles due to the proven efficacy of immunizations, especially for poorer households^52^. However, despite healthcare needs for people living with HIV (PLWHIV) being subsidized, studies have reported that PLWHIV still incurred CHE at high rates^53,54^. For PLWHIV, incidence of CHE was majorly driven by inpatient admissions, income levels, and ethnicity.

Even minor health needs, such as wound dressings associated with more significant illnesses/ conditions such as burns, accidents and other pathological conditions drive CHE in a large percentage of patients. The respondents having a mean monthly earning of 50,000 naira and dressings costs ranging between N9000 ($5.4) - N27000 ($16.2) per week ^55,56^.

## Coping Mechanisms

This review reveals that CHE is still incurred in the management of a majority of health care needs. In tandem with widespread multidimensional poverty, this necessitated the adoption of coping mechanisms to access healthcare. Many healthcare users resort to using personal savings, selling assets and obtaining loans for this purpose^57^. It is also quite common for users to seek financial support from friends and family. Another common coping mechanism is the patronage of patent medicine vendors and informal healthcare givers. These individuals (patent medicine dealers, chemists and traditional healers) offer a wide range of services ranging from the dispensing of medicines, wound dressing, consultation, obstetrics and gynecology and even minor surgery^58^. Despite the known dangers, high patronage (60% of individuals) is driven by affordability compared with orthodox options^59^. Interestingly, self-medication was another reported coping mechanism aimed at reducing health costs^60^. Finally, there was a variance in the coping mechanism employed by wealth class, with the least poor cutting down on other expenses and the poorest who do not have disposable income opting to borrow^61^.

## Forgone Healthcare

Despite the existence of numerous coping mechanisms, access to healthcare still falls out of the reach of some users due to financial barriers. Especially among the poorest quintile, these options are sometimes not available to then or enough to pay for healthcare. Consequently, these users have to forgo healthcare. This is perhaps the greatest challenge of the financial risk associated with healthcare, as common outcomes include emergency hospitalization (that inflates costs and leads to impoverishment), health complications, reduced quality of life and even death^62^. These outcomes typically present within 2–8 years. However, although this subject has been extensively explored in developed countries, the dearth of literature on this niche in Low-and Middle-Income countries (LMICs) is described by Kabembo as a pervasive void^63^. Nonetheless, the extant literature provides meaningful insight into the scope of this challenge.

In Nigeria, studies show that between 30% and 60% of healthcare users in different cohorts had to forgo healthcare, with financial hardship cited as the major limitation^64,65^. However, the prevalence of forgone healthcare was much lower for people who used reproductive, maternal, newborn and child health (RMNCH) services, even during the pandemic^66^. This outcome can be attributed to the subsidization of RMNCH services by domestic and international partners confirming the impact of public funding in shielding against financial hardship^67,68^. This subsidy ensures that the position of Ujah and colleagues, that food insecurity is correlated with forgone healthcare among peripartum women did not hold in a country where 75% of pregnant women face food insecurity^69,70^.

Compared with previously published works on the financial risk faced by Nigerians, CHE and IHE have increased in all regions and across all health groups^71,72^. This can be attributed to the devaluation of the naira and the exit of numerous multinational pharma companies due to unfavourable economic and political conditions. These factors have also increased multidimensional poverty, in the nation which is a major determinant of both CHE and IHE. Given the current realities, there is need to discuss the protective mechanisms that were designed to protect against financial risk.

## FRP Interventions In Nigeria

Poor financing mechanisms challenge UHC efforts as the Minister of Health Dr. Ehanire emphasized during the World UHC day in 2022^73^. He called for a transition from out-of-pocket (OOP) to other options explored by Uzochukwu and colleagues^26^. OOP remains the major cause of financial risk associated with healthcare. Although there are other sources of funds for healthcare, OOP still makes up 69% of health payments with contributions from the NHIA and the government accounting for 30% and less than 1% contributed by indigenous non-governmental organizations (NGO) and private firms^14^ Although significant donor funds contribute to health financing in Nigeria, those funds are used for targeted interventions and has little effect on the overall health system of the country^74^. This leaves tax-based funding and pooled payments as the major sources of funding available to the country^75^.

## Tax- Based Funding

Tax-based funding is suggested to be the best source of funding for achieving UHC especially in LMICs^76^. Studies show a positive correlation between utilization of tax revenue and progress towards UHC^77^. According to Aregbeshola^76^, it is difficult for countries with a large proportion of their citizens in the informal work sector or in vulnerable groups to ensure total coverage via a voluntary contributory social insurance scheme. Studies highlight challenges with reaching rural populations, income instability within the informal sector and fragmented insurance schemes as reasons for failure of SHI^78–80^. However, Nigeria has one of the lowest tax revenue outputs, even when compared to other LMICs^77,81^. This, in conjunction with other abysmal financial policies allow per capita spending of $11.2, which falls short of the expected minimum of $86 per capita on health, thereby crippling such interventions^82^. Despite official commitments such as the Abuja declaration, government health expenditure as a percentage of total expenditure is approximately 0.65%. Therefore, an enormous gap remains to be scaled if public financing as a tool is significant in the journey towards UHC. Evidence from Brazil, Thailand and other LMICs show how efficient implementation of a tax-based noncontribution system can lead to the attainment of UHC^83^. By increasing budgetary allocation for healthcare to 16%, Zambia has been able to abolish user fees in all PHCs, rural and peri-urban areas, leading to a reduction in financial risk involved^84^.

## Social Health Insurance Program

Pooled payments remain the major remedy to OOP, distributing health costs among the members of the pool who pay a premium. In Nigeria, available pooled payment methods include the social health insurance (national health insurance and state health insurance), community-based health insurance and private health insurance (PHI). These are operated by various accredited health maintenance organizations (HMOs) that mediate between service providers and users^85^. In 2021, the Minister of health emphasized the importance of widespread enrollment in the national health insurance scheme. Sadly, many Nigerians erroneously believe that the NHIA is for federal government workers or urban dwellers only. This has contributed to limited uptake even in the face of debilitating OOP. Workers on the federal payroll account for only 5% of the population as an estimated 76.7% of Nigerians are employed in the informal sector^86^. However, the institution of the Group Individual and Family Social Health Insurance (GIFSHIP) in the NHIA act of 2021 extends this coverage to the informal sector with similar insurance plans^87^. However, even among NHIA enrollees, utilization is low, as studies report that 70% of these users still finance health needs independently^88^. This is due to many drawbacks such as poor service delivery, limited scope of service coverage, frequent stockouts and low accountability in resource management.

Unlike the NHS, users still make payments at the point of service for up to 10% of total costs^89,90^. This finding contributes to the evidence showing that although the NHIA offered FRP in some ways, it did not wholly protect against CHE with many studies reporting CHE levels of up to 50%^91^. Furthermore, Uduu^92^ suggested that the non-inclusion of PHCs as service points for HMO marginalizes people living in rural areas. To access insurance services, they would need to incur transportation costs, which many users in that demographic cannot afford alongside other indirect costs, e.g., hours of work missed.

Owing to the limitations of the NHIA and the continued need to reach the underserved, several states adopted the state health insurance scheme with Lagos State being the pioneer in 2015^93^. As of 2019, only 18 states had adopted this initiative^92^. Although they have shown promise to overcome the limitations of low adoption and cost-effectiveness, they soon ran into the same bottlenecks as the national scheme^94,95^. Corruption and fraud remain the major challenges to the actualization of UHC through FRP. These reports highlight the poor quality of drugs dispensed for service users in private institutions and public health facilities. Similarly, Okoro and others^96^ reported unethical practices related to prescribing practices, patient care, counselling and adherence to NHIA guidelines. All these are directed towards maximizing profits from HMOs.

## Community Based Health Insurance Programs

There are no data on the number of CBHI programs in the country. However, available studies show its presence in the majority of states. With a focus on rural areas, this intervention was designed to reduce inequality in coverage between rural and urban areas^97^. These schemes have characteristic lower premiums (approximately N5000 per year or $3), as they benefit from government and private subsidies^98^. Usually, service users are attached to the PHCs in those communities. While its effectiveness in protection against financial risk has been proven, its challenges are unique from those of the NHIA and SHI. First, the majority of the populace is unaware of their existence which affects enrollment and pooling. Second, this intervention serves the poorest quintile, some of which are not able to afford or unwilling to pay a premium of N2000 ($1.2) per annum. Furthermore, these schemes have no defined operational designs and their structure varies widely across regions, thus affecting evaluation processes^99^. Odeyemi^100^ also reported that CBHI in Nigeria suffers neglect from the government which has marred its success. Other factors including the deplorable condition of PHCs, the unmet targets of HMOs and the unethical practices of healthcare providers, have contributed to the underachievement of CBHI^101^.

## Private Health Insurance

Perhaps the most efficient financial risk intervention is private health insurance of which 94% is employer-based^102^. However, owing to expensive premium rates, adoption is limited. Nonetheless, private HMOs offer wider coverage than do NHIA, which focus on basic or essential health services. Other advantages include the use of ambulances systems, inpatient services and more efficient administration^103^.

## Achieving UHC Through FRP – The Way Forward

As discussed in the previous section, there is a need to shift focus from pooled payments to tax-based funding. This has been shown to be a more sustainable approach and also remedy the shortcomings of pooled payments like poor coverage of informal sectors and a fragmented service provision. This is a call for the initiation of legislative and political discourse on the feasibility of this transition in the Nigerian context, and the design of the optimal framework for its implementation^104^.

However, until said mechanisms can be devised, it is necessary to improve the pool of funds for health payments. To achieve this goal, the government would need to increase the number of enrollees fueled by targeted awareness campaigns. The various options for social insurance and the need for affordable healthcare should be focal points of these campaigns. The thrift system should be adopted for the CBHI in Nigeria^105^. A major advantage of this method is familiarity and trust, as thrift networks such as “Esusu” and “Ajo” are commonly used within the target demographic to serve other needs, such as food and education^106^. It has also been proven to create a significant pool of funds through cyclical payments. However, it remains to be studied if this system can handle the multiple and continuous payouts which is characteristic of insurance systems. It is important that feasibility studies be carried out to identify the cross-regional variations that will affect implementation of such programs.

Secondly, the sustainability of health insurance schemes especially in regions where OOP is required at points of service delivery, is dependent on the willingness to pay (WTP) and by extension, the CTP of the users^107,108^. This WTP is defined as the maximum amount of money that service users are ready to pay to access a health service^109^. However, seeing that intent does not translate into payments, CTP has proven to be a more precise determinant of sustainability^110^. In Nigeria, premium payments for a family of five cost N60,000 ($36 at current rates) which is unaffordable for some families. Philanthropists and NGOs should be encouraged to take advantage of the GIFSHIP program of the NHIA to extend coverage to underserved individuals^90^. Another intervention could be the introduction of full subsidies for direct health costs for high-mortality, high-impact diseases such as malaria in high-risk groups (children under 5years), which would significantly improve financial protection outcomes. While it may seem daunting for NCDs such as cancer, excising taxation on tobacco products and other potential carcinogenic products can serve as a source of funding for subsidized treatment and other harm reduction efforts ^111,112^.

It is important to retrain healthcare service providers within the insurance system^96^. Frequent monitoring and evaluation should be carried out at accredited service points to improve quality. This would require the development of effective information systems, especially in rural and peri-urban areas. A whistleblower system can prove to be advantageous in reporting the incidence of fraud. Monitoring and evaluation should also be extended to outcomes such CHE. Neglected metrices such as forgone healthcare and coping mechanisms should become central to reports.

Service providers need to optimize the costs of running the program. Improved efficiency and cost effectiveness can be achieved only by the collaboration of all major stakeholders. The duplicity of roles and other administrative costs need to be reduced to release more funds into the payment pool. Quality improvement methodologies such as lean thinking and six sigma need to be applied to optimize these processes^113^. There is a need for synergy at all levels of healthcare for the actualization of FRP. Healthcare providers, HMOs and the government must work in tandem to achieve their common goals.

In summary, corruption and the lack of political will which lies at the heart of every failed policy in the country, must be addressed if financial protection is to be achieved^114^. As long as Nigeria ranks low in the corruption perception index, the incongruity that plagues the health sector will continue to undermine efforts towards UHC. In the same vein, much progress will be made if political leaders prioritize UHC and see it as a need for their constituents. This should be the thought of the electorates when selecting leaders. Therefore, politicians need to improve in the transparency, efficiency and implementation of adopted policies if UHC is to be achieved^115^.

## Conclusion

Despite multiple commitments towards actualizing UHC, Nigeria is still afar off in this journey. Although measurable progress has been made in improving access to healthcare and service coverage, many of these service users are still exposed to financial risk. This study shows the neglect of FRP as health seekers face CHE and IHE across disease-specific and region-specific cases. The concurrent economic decline and exit of pharmaceutical companies has compounded outcomes. In extreme cases, users have to forgo healthcare or adopt coping mechanisms. FRP is only observed in donor-funded interventions for diseases such as HIV/AIDS and vaccine preventable conditions such Measles and mumps. Donor funded interventions are not sustainable, which necessitates improvements in pooled payments and government funding. This review has examined the case for both interventions and provided recommendations that will improve financial risk outcomes and set Nigeria on the path towards achieving UHC.

## Data Availability

No datasets were generated or analysed during the current study.
